# *Cordylobia anthropophaga* Myiasis Mimicking Hyperproliferative Skin Disorder in Traveler Returning from Sub-Saharan Africa

**DOI:** 10.3390/tropicalmed8110505

**Published:** 2023-11-20

**Authors:** Lidija Popović Dragonjić, Andrija Jović, Irena Janković, Jelena Miladinović, Aleksandar Ranković, Maja Cvetanović, Relja Beck, Dinko Novosel, Thomas Pape, Pavle Banović

**Affiliations:** 1 Department of Infectious Diseases and Epidemiology, Faculty of Medicine Niš, University of Niš, 18000 Niš, Serbia; drarankovic@gmail.com; 2 Clinic for Infectology, University Clinical Center Niš, 18000 Niš, Serbia; miladinovic.jelena.ni@gmail.com (J.M.); maja.majcan@hotmail.com (M.C.); 3 Clinic for Dermatology, University Clinical Center Niš, 18000 Niš, Serbia; dr.andrijajovic91@gmail.com; 4 Department of Surgery and Anesthesiology and Reanimatology, Faculty of Medicine Niš, University of Niš, 18000 Niš, Serbia; irena.jankovic@medfak.ni.ac.rs; 5 Clinic for Plastic Surgery, University Clinical Center Niš, 18000 Niš, Serbia; 6 Department for Bacteriology and Parasitology, Croatian Veterinary Institute, Savska Cesta 143, 10000 Zagreb, Croatia; relja.beck@gmail.com; 7 Department for Pathological Morphology, Croatian Veterinary Institute, Savska Cesta 143, 10000 Zagreb, Croatia; dinko.novosel@gmail.com; 8 Natural History Museum of Denmark, Science Faculty, University of Copenhagen, 2100 Copenhagen, Denmark; tpape@snm.ku.dk; 9 Clinic for Lyme Borreliosis and Other Tick-Borne Diseases, Department of Prevention of Rabies and Other Infectious Diseases, Pasteur Institute Novi Sad, 21000 Novi Sad, Serbia; 10 Department of Microbiology with Parasitology and Immunology, Faculty of Medicine in Novi Sad, University of Novi Sad, 21000 Novi Sad, Serbia

**Keywords:** *C. antropophaga*, returning traveller, myiasis, pathohistology, Serbia

## Abstract

Myiasis is one of the most common skin diseases found in travelers returning from tropical and subtropical regions, where humans living in or visiting the African continent are most commonly infested by *C. anthropophaga* during the rainy season in regions with a warm climate. Here, we present a case of furuncular myiasis caused by *C. anthropophaga* in a Serbian patient returning from temporary work in Kenya, where the initial histology of skin lesion mimicked hyperproliferative skin disorder.

## 1. Introduction

Myiasis is a condition caused by an infestation of live vertebrates (humans/animals) with fly larvae that feed on tissues, body fluids, or intestinal content [1]. Although various body parts can be affected by fly larvae infestation, skin myiasis is most common, developing as one of three clinical forms (i.e., furuncular, migratory, and wound myiasis) [2]. Furuncular myiasis is a skin infestation in the form of a small, raised, red, bite-like lesion that may grow into an erythematous, tender nodule [3]. It is most often caused by infestation with *Cordylobia anthropophaga*, *Dermatobia hominis*, *Wohlfahrtia vigil*, and *Cuterebra* spp. [4]. *Cordylobia anthropophaga* and *Dermatobia hominis* are the most common vectors of myiasis in Africa and the Neotropics, respectively [5]. *Wohlfahrtia vigil* and *Cuterebra* spp. are the most common causes of furuncular myiasis in North America [6].

*Cordylobia anthropophaga* (tumbu fly) oviposits in dry sand contaminated by urine or feces or on exposed clothing, and always in the shade [7]. Most human infestations occur on non-exposed parts of the body, such as the trunk, as opposed to the face, arms, and legs, which are less frequently affected. First, instar larvae hatch after a few days and enter the skin of a person or animal they may opportunely come into contact with. The larva develops via three instars under the skin, resulting in a furunculoid skin lesion with a breathing hole for the larval posterior spiracles on the surface. When mature, the 3rd instar larva leaves the host, drops to the ground, and buries itself to pupate. The adult fly will usually emerge in 10–11 days [7,8].

In Europe, cases of cutaneous myiasis due to *C. anthropophaga* (tumbu fly) are observed in travelers returning from sub-Saharan parts of tropical Africa, although a few cases have been incriminated to have been acquired locally [9,10]. Cases may go undiagnosed or mistaken for some other skin conditions (e.g., furunculosis, skin malignancies) [11,12,13,14]. Here, we present a case of furuncular myiasis caused by *C. anthropophaga* in a Serbian patient returning from temporary work in Kenya.

## 2. Materials and Methods

On 12-XI-2022, a 44-year-old man returning to Serbia from temporary work in Mombasa, Kenya, noticed skin alteration on the abdomen and right hand, along with an occasional “tingling” sensation. He was otherwise healthy, without comorbidities and any familial burden of hereditary diseases. He thought that those complaints were the consequences of ingrown hairs. During his stay in Kenya, he dried his clothes on a string outdoors in spite of warnings from nearby residents. He was admitted and treated at the Infectious Disease Clinic of the University Clinical Center Niš (Niš, Serbia). After the removal of fly larvae, samples were forwarded for morphological identification to Pasteur Institute Novi Sad (Novi Sad, Serbia) and for molecular analysis to the Department for Bacteriology and Parasitology of Croatian Veterinary Institute (Zagreb, Croatia). On the same day, blood was sampled for routine laboratory analyses (i.e., complete blood count, biochemical analyses, inflammatory markers, coagulation status, etc.). Morphological investigations for species identification were performed using an Optika SLX-1 stereomicroscope (Optika S.r.l., Ponteranica, Italy).

DNA was extracted from 25 mg of parasite tissue using the QIAcube automated DNA isolation system (Qiagen, Hilden, Germany) and QiaAmp DNA mini QIAcube Kit, according to the manufacturer’s instructions (Qiagen) in the final volume of 200 μL. Amplification of the barcode region of COI was performed using the primers LCO1490 and HCO2198 [15]. PCR reaction mixtures of 20 μL were prepared containing 10 μL G2 GOTaq mastermix (Promega, Madison, WI, USA), 8.2 μL of DNase/RN ase-free distilled water (Promega), 0.4 μL of 10 pmol/μL each primer, and 1 μL of the sample. Positive and negative controls, including extraction controls, were included in all amplifications. Amplification results were evaluated by capillary electrophoresis with QIAxcel (Qiagen, Hilden, Germany) using the QIAxcel DNA Fast Analysis kit, alignment markers (DNA QX Alignment Marker 15 bp/3 kb), and marker sizes (QX DNA Size Marker 50–3000 bp).

The amplified sample was purified using EXOSAP-it^®^ (USB^®^ Products Affy Inc., Cleveland, OH, USA) according to the manufacturer’s instructions and sequenced in both directions by Macrogen Inc. (Amsterdam, The Netherlands) using the same pair of primers. Sequences were assembled using the SeqMan Pro software v12.2.0 (82),421, edited with the EditSeq software (Lasergene, DNASTAR, Madison, WI, USA), and compared with available sequences in GenBank^®^ using Basic Local Alignment Search Tool (BLAST^®^).

A total of 22 sequences of segment 651 nb of the COX1 gene of the mtDNA were analyzed. A total of 6 sequences belonging to *C. anthropophaga* were found using the blast tool in NCBI (accessed 8 November 2023). Sixteen additional sequences belonging to phylogenetically related species were included in the dataset for phylogenetic analysis: *Hemigymnochaeta unicolor* (1), *Pachychoeromyia praegrandis* (1), *Lucilia sericata* (5), *Lucilia cuprina* (3), *Calliphora nigribarbis* (1), *Calliphora vomitoria* (2), *Calliphora quadrimaculata* (1), *Calliphora uralensis* (1), and *Calliphora lata* (1).

**Figure 1:**
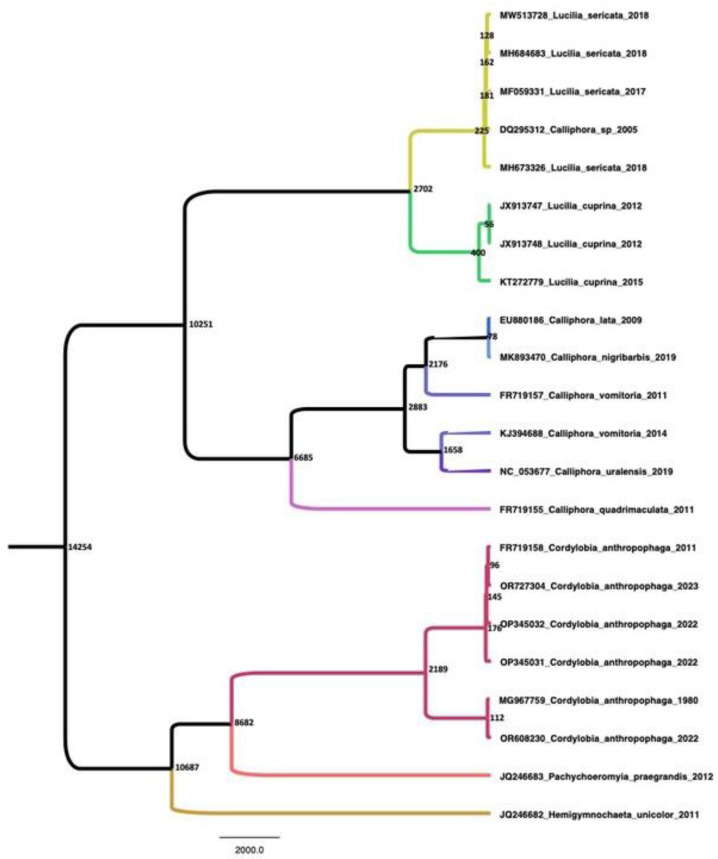
MCA tree. Node age values are presented by the nodes where the posterior probability value was PP > 0.9.

Taxon names used in Figure 1 consist of the following information: “Genbank acces-sion number_Species name_year of isolation”. Sequences were aligned using the Muscle algorithm in AliView software v1.28 with minor modifications, as the sequence contains some stop codons as a result of an error during assembly. Bayesian phylogeny was calculated using the software package BEAST v1.10.4. The program was run using the Markov Chain Monte Carlo length until effective sample size values were over 200. The calculation was performed using the codon model Yang 1996 (YANG96) [16,17], uncorrelated relaxed lognormal molecular clock [16], and the Coalescent Bayesian Skyline model. BEAST .log files were analyzed in Tracer v1.6. The selected tree file was compiled in Tree-Annotator from the BEAST package, and the Most Common Ancestor (MCA) tree was constructed in FigTree v1.4.3 [18].

Since the furuncular form of myiasis can be associated with skin cancers [12,14], a skin biopsy was performed on the abdomen to rule out the possibility of malignant alteration (30-XI-22). Biopsy samples were forwarded to the Pathology and Pathological Anatomy Center of the University Clinical Center Niš (Niš, Serbia). After fixation in 4% buffered formalin, samples were embedded in paraffin, and 4 micrometer-thick sections were stained with hematoxylin and eosin.

## 3. Case Presentation

**Figure 2:**
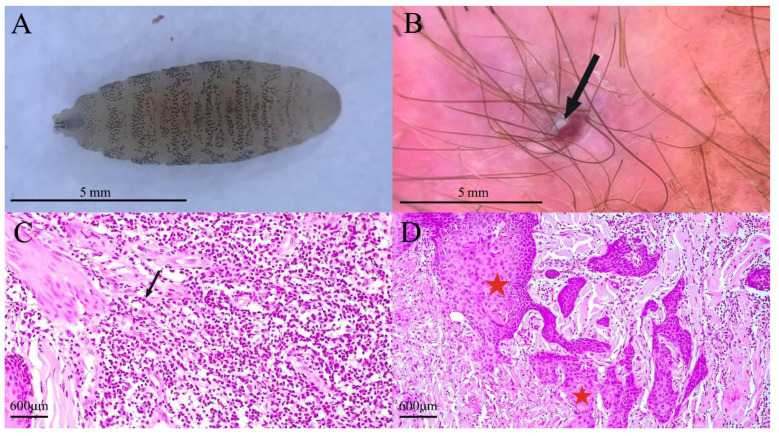
(**A**) *Cordylobia anthropophaga* larva pulled out from the righand lesion. Multiple brownish cuticular spines are observed on all body segments. (**B**) Dermoscopy of the lesion on the dorsal part of right hand. Small whitish part of larva surfacing (black arrow) at the central pore (4gen Dermlite DL4 coupled with iPhone 13, 10× magnification). (**C**) Histopathological findings of skin biopsy, dermis. Polymorphic inflammatory infiltrate made of numerous eosinophils, neutrophils, and lymphocytes (arrows) (H&E, original magnification 200×). (**D**) Histopathological findings of skin biopsy, epidermis, and dermis. Proliferation of squamous cells of the epidermis (red star) and its extending (red star) into the reticular layer of the dermis showing no mitotic activity (H&E, original magnification 100×).

The patient returned to Niš on 19-XI-22, and three days later, his skin changes began to resemble an abscess. From 22-XI to 27-XI-22, the patient noticed swelling of both skin lesions with the appearance of surrounding redness. On 28-XI-22, the abdominal skin alteration increased significantly in size, and the patient tried to squeeze out the contents, successfully removing one live and mobile maggot, approximately 5 mm in length (Figure 2A). Physical examination of the right arm and abdomen revealed furunculous lesions with reactive erythema and swelling (4 cm and 3 cm in diameter, respectively) (Figure 2B). A second fly larva (approximately 4 mm in length) was observed and removed from the right-hand lesion during the admission. On the following day (29-XI-22), Albendazole 400 mg b.i.d. and Clindamycin 300 mg t.i.d. therapy was initiated and continued for 5 days.

The larva showed a cylindrical body and 12 visible segments diffusely covered in spines directed posteriorly, which corresponds with the genus *Cordylobia*. The posterior spiracles open through three sinusoidal openings with a weakly sclerotized peritreme, which corresponds to the third larval instar of *Cordylobia anthropophaga* [13].

Morphological identification was further confirmed via the sequencing of a 683-bp portion of the COI gene. The obtained sequence was identical to the *C. anthropophaga* sequence deposited from Kenya, Nairobi (Acc. No. MG67759) and shared 97% similarity with sequences from Cameroon (FR719158) and Gambia (OP345031). A sequence was deposited in the GenBank database under accession number OR608230.

The sequenced isolate of *C. anthropophaga* extracted from the Serbian patient OR608230 shows a strong phylogenetic relationship with sequence MG967759 isolated in 1980 in Kenya (Figure 1). The common ancestor of sequences OR608230 (Serbia, 2022) and MG967759 (Kenya, 1980) is estimated to have existed 112 years ago. There is a second branch consisting of four sequences—FR719158, OR727304, OP345032, and OP345031—isolated in Cameroon, Senegal, Poland, and Gambia, respectively. These sequences originate geographically from the western part of Africa, in contrast to the other branches, where the sequences originate from the eastern part of Africa.

During the therapy, the swelling and hyperemia gradually receded, and the skin changes became smaller in diameter and duller in color. Two days after the end of the therapy (5-XII-22), the edema disappeared, and only a dull red area was visible at the site of the former swelling (i.e., the lesion was at skin level with a diameter of 1 cm). There were no new skin lesions that would indicate a still active infestation. A month later, the next check-up was performed and no signs of inflammation were detected at the previous infestation sites.

Among routine laboratory blood results, only the levels of creatine kinase were found to be elevated, probably as a consequence of physical work reported by the patient. All routine laboratory findings are presented in Supplementary Table S1.

Histological analysis of skin biopsy samples showed squamous cell proliferation in the epidermis (Figure 2C), while the dermis was infiltrated with polymorphic inflammatory infiltrate (Figure 2D). No remnants of fly larvae were found.

## 4. Discussion

Myiasis is one of the most common skin diseases found in travelers returning from tropical and subtropical regions. The survival and dissemination of myiasis-causing larvae is highly dependent on environmental factors [19]. Humans living in or visiting the African continent are most commonly infested by *C. anthropophaga* during the rainy season in regions with a warm climate [20], where furuncular myiasis is the most common type. Children are more frequently infested than adults due to thinner skin and the absence of an acquired immune response, which is developed only after repeated exposures [20]. The larvae penetrate the undamaged skin of the host, who has either been in contact with the soil or was wearing contaminated clothing. Patients usually feel no disturbance when a larva penetrates the skin, and in most cases, returning travelers do not suspect maggots as the cause of their ailments [13]. Although myiasis in the Serbian population has previously been described, all reported cases were autochthonous and not related to travel as a risk-related activity [21,22].

Covered body parts are considered the most vulnerable for *C. anthropophaga* infestation since the fly lays its eggs on wet clothes that are left out to dry [8]. In the case described here, a covered part of the body was indeed infested since one larva was found in the abdominal skin, while a second one was found in an exposed body part (i.e., the hand). Laboratory test results in cutaneous myiasis are most often unremarkable, while in cases of chronic infestation and/or multiple infestations, eosinophilia and elevated levels of C-reactive protein and IgE are observed [20]. Blood count and biochemical analyses of our patient yielded results within the reference range, especially considering that there was no eosinophilia. This finding is in agreement with the fact that the infestation did not take on the characteristics of systemic inflammation and that the pathophysiological process remained local (i.e., only in the skin).

Persons at risk for *C. anthropophaga* infestation are instructed to iron linen and clothes after washing, as well as to avoid drying laundry outside and near the ground, and in particular, not in the shade. Wearing clothes that have been put in the shade on the ground should be avoided. Nevertheless, the main prevention measures for *C. anthropophaga* infestation are careful personal hygiene and the application of insect repellents to eliminate flies from the work and living space [23]. Accordingly, it is especially important for residents of non-endemic countries who travel to tropical and subtropical regions to familiarize themselves with the prevention and clinical presentation of myiasis [23,24]. In the case described here, all of the mentioned preventive measures were occasionally violated, mainly due to a lack of knowledge. This case highlights the need to consider cutaneous myiasis in a patient returning from tropical and subtropical regions with abscess-like bumps, in addition to other diagnoses. Also, close contacts of a returned traveler infested with *C. anthropophaga* should be instructed to perform self-examination since fly eggs or newly hatched larvae can be transmitted to other humans by contaminated clothing [25].

Diagnosis of furuncular myiasis in endemic countries can be made by experienced clinicians or infectious disease specialists solely based on clinical findings. In non-endemic territories, several diagnostic tools can aid the clinician via the process of differential diagnosis, such as dermoscopy (for the morphological evaluation of the lesion and parasite), ultrasound (allows for the differentiation of a simple abscess from furuncular myiasis), and color Doppler sonography (visualizes the continuous movement of larvae internal fluids) [20,26].

Pathohistological findings of skin biopsy samples from sites affected by myiasis are not common [27]. Here, we found pseudocarcinomatous hyperplasia, with thin and bland-appearing epithelial proliferates tending to anastomose. Mitosis activity of cells was rare and without atypical mitoses. The significance of this finding is that pseudocarcinomatous hyperplasia can be difficult to distinguish from other hyperproliferative skin disorders, especially squamous cell carcinoma. Socio-epidemiological survey, entomological examination of larva, and clinical recognition of possible myiasis are essential to avoid misdiagnosis.

Skin myiasis treatment involves local and systemic measures. In most patients, it is necessary to remove the larva at the time of examination or diagnosis. The literature describes many different methods of removing larvae from furuncles [28], including the use of lateral pressure or suffocation of the larva by closing the pore with medical liquid paraffin/mineral oils/animal fat. Surgical incision is the most recommended intervention since it prevents the rupture of the larva and the resulting granulomatous reaction [3]. Positions about systemic administration of ivermectin and albendazole in the treatment of furuncular myiasis are divided since a dead larva inside the skin can trigger a consequent inflammatory reaction [20]. Since secondary infections are possible, concomitant treatment with antibiotics is recommended [29,30].

So far, too few sequences are known to draw more precise conclusions, but our results indicate that there are two phylogenetically distant haplogroups of *C. anthropophaga* of the western (FR719158, OR727304, and OP345031) and eastern (MG967759) tropical regions of Africa.

## 5. Conclusions

Up to our knowledge, this is the first case of *C. anthropophaga* myiasis imported to southeastern Europe. Myiasis caused by *C. anthropophaga* can be easily diagnosed in non-endemic territories if an adequate epidemiological anamnesis is applied, since the spectrum of diseases that can cause furunculoid skin lesions is large. The appearance of skin changes and the geographical location of the patient’s travel are sufficient to suspect myiasis and apply other diagnostic and therapeutic procedures, such as dermoscopy, microbiological analysis, and antiparasitic and antibiotic therapy.

## Data Availability

Data are contained within the article and supplementary material.

## Supplementary Materials

The following supporting information can be downloaded at https://www.mdpi.com/article/10.3390/tropicalmed8110505/s1. Table S1. Laboratory findings in the patient with *C. anthropophaga* myiasis.
