# Supplementary material for: Cordylobia anthropophaga Myiasis Mimicking Hyperproliferative Skin Disorder in Traveler Returning from Sub-Saharan Africa
Source: Trop Med Infect Dis. 2023 Nov 20;8(11):505. doi: 10.3390/tropicalmed8110505 (PMC10674998; doi:10.3390/tropicalmed8110505)
Supplement: Supplementary file 1 [file tropicalmed-08-00505-s001.zip › tropicalmed-2673897-supplementary.pdf]

Supplementary Table S1 – Laboratory findings in the patient with *C. anthropophaga* myiasis

Parameter of interest	Result	Status	Ref.values
Leukocyte count (10^9/L)	5.8	WNL	4.0-9-0
Lymphocytes (%)	41.02	WNL	20.00-50.00
Neutrophiles (%)	44.32	WNL	40.00-70.00
Monocytes (%)	9.03	WNL	1.00-10.00
Eosinophiles (%)	4.53	WNL	0.00-6.00
Red Blood Cell Count (10^{12}/L)	4.96	WNL	4.30-5.80
Hemoglobin (g/L)	148	WNL	120-180
Hematocrit (L/L)	0.451	WNL	0.410-0.560
Platelet Count (10^9/L)	179	WNL	120-380
Glucose (mmol/L)	5.1	WNL	3.9-6.1
Urea (µmol/L)	6.2	WNL	2.5-7.5
Kreatinine (µmol/L)	93.8	WNL	53.0-115.0
Total bilirubin (µmol/L)	11.4	WNL	5.0-21.0
Direct bilirubin (µmol/L)	2.7	WNL	0.0-3.4
Total proteins (g/L)	75	WNL	62-81
Albumin (g/L)	45	WNL	35-52
C-reactive proteine (mmol/L)	1.8	WNL	0.0-5.0
Cholesterol (mmol/L)	4.15	WNL	3.90-5.20
Triglycerides (mmol/L)	0.80	WNL	0.70-1.70
Sodium (mmol/L)	138	WNL	135-148

Potassium (mmol/L)	4.2	WNL	3.5-5.5
Chlorides (mmol/L)	104	WNL	98-108
Iron (µmol/L)	21.4	WNL	12.5-32.2
Aspartate aminotransferase (U/L)	16	WNL	10-37
Alanine aminotransferase (U/L)	17	WNL	10-42
Alkaline phosphatase (U/L)	48	WNL	30-120
Gamma glutamyl transpeptidase (U/L)	22	WNL	0-55
Alpha amylase (U/L)	46	WNL	28-100
Creatine kinase (U/L)	200	↑	0-195
Creatine kinase-MB (U/L)	6	WNL	0-24
Rheumatism factor (IU/mL)	7.10	WNL	0.00-14.00
Prothrombin time International normalized ratio	1.03	WNL	0.80-1.20
Prothrombin time (%)	95	WNL	75-120
D-dimer (ng/mL)	135	WNL	0.0-250
